# Effects of Silica Nanoparticles on the Piezoelectro-Elastic Response of PZT-7A–Polyimide Nanocomposites: Micromechanics Modeling Technique

**DOI:** 10.3390/polym16202860

**Published:** 2024-10-10

**Authors:** Usama Umer, Mustufa Haider Abidi, Syed Hammad Mian, Fahad Alasim, Mohammed K. Aboudaif

**Affiliations:** 1 Advanced Manufacturing Institute, King Saud University, P.O. Box 800, Riyadh 11421, Saudi Arabia; 2 Industrial Engineering Department, College of Engineering, King Saud University, P.O. Box 800, Riyadh 11421, Saudi Arabia

**Keywords:** PZT–polyimide composite, silica nanoparticle, piezoelectro-elastic properties, interphase, micromechanics modeling

## Abstract

By using piezoelectric materials, it is possible to convert clean and renewable energy sources into electrical energy. In this paper, the effect on the piezoelectro-elastic response of piezoelectric-fiber-reinforced nanocomposites by adding silica nanoparticles into the polyimide matrix is investigated by a micromechanical method. First, the Ji and Mori–Tanaka models are used to calculate the properties of the nanoscale silica-filled polymer. The nanoparticle agglomeration and silica–polymer interphase are considered in the micromechanical modeling. Then, considering the filled polymer as the matrix and the piezoelectric fiber as the reinforcement, the Mori–Tanaka model is used to estimate the elastic and piezoelectric constants of the piezoelectric fibrous nanocomposites. It was found that adding silica nanoparticles into the polymer improves the elastic and piezoelectric properties of the piezoelectric fibrous nanocomposites. When the fiber volume fraction is 60%, the nanocomposite with the 3% silica-filled polyimide exhibits 39%, 31.8%, and 37% improvements in the transverse Young’s modulus $E_{T}$, transverse shear modulus $G_{TL}$, and piezoelectric coefficient $e_{31}$ in comparison with the composite without nanoparticles. Furthermore, the piezoelectro-elastic properties such as $E_{T}$, $G_{TL}$, and $e_{31}$ can be improved as the nanoparticle diameter decreases. However, the elastic and piezoelectric constants of the piezoelectric fibrous nanocomposites decrease once the nanoparticles are agglomerated in the polymer matrix. A thick interphase with a high stiffness enhances the nanocomposite’s piezoelectro-elastic performance. Also, the influence of volume fractions of the silica nanoparticles and piezoelectric fibers on the nanocomposite properties is studied.

## 1. Introduction

Piezoelectric-fiber-reinforced polymer composites are used for various engineering applications, such as in clean energy harvester devices from environmental sources and in vibration control, structural health monitoring, and structural morphing [1,2,3,4]. The improved mechanical flexibility, good stiffness/strength-to-weight ratio, reliability, and tailorable properties have made piezoelectric fibrous composites more favorable for the abovementioned applications as compared to neat piezoelectric materials [4,5,6,7,8]. Nevertheless, advances in electro-mechanical technologies have led to fast growth in the demand for piezoelectric fibrous composites with better functionalities. So, the design of new piezoelectric composites is an important issue for researchers and scientists.

The concept of using nano/micro-hybrid reinforcements in polymer composites has emerged for the sake of improving their multifunctional properties. In these composites, nano-sized reinforcements such as graphene nanoplatelets, carbon nanotubes (CNTs), and silica nanoparticles are introduced alongside traditional micron-sized fibers [8,9,10,11,12,13]. For example, Cui et al. [13] observed that carbon fiber–silica nanoparticle composites have better performance regarding interlaminar shear strength, impact strength, and flexural strength compared with that of carbon fiber composites. Hwayyin et al. [14] indicated an enhancement in the mechanical properties of carbon fiber–polyester composites at different weights of nano-silicon dioxide. The outcomes showed an increment in the tensile stress of 11.45% after an increase in the nanoparticle content from 0.16% wt. to 0.2% wt. [14]. Zheng et al. [15] studied the influence of silica nanoparticles on the mechanical properties of glass-fiber-reinforced epoxy composites. It was observed that the increase in the silica nanoparticle content yields an enhancement in the tensile modulus and compression strength. Gang et al. [16] reported that an increase from 0% to 5% of the nanoparticle volume fraction yielded an enhancement in the tensile modulus of a carbon fiber–silica nanoparticle–polyimide composite from 2475 MPa to 2780 MPa. Tang et al. [17] measured tensile properties in the transverse direction, interlaminar shear strength, and the mode I and mode II interlaminar fracture toughness of carbon fiber composites with 10 wt% and 20 wt% silica nanoparticles dispersed into epoxy. The transverse tensile properties and mode I interlaminar fracture toughness were improved by increasing the silica nanoparticle content in the epoxy [17].

Such a concept has been employed to study the advantages of nanofiller-containing matrices in improving the equivalent properties of piezoelectric fibrous composites. Keramati et al. [12] analyzed a nanocomposite in which BaTiO_3_ fibers were placed inside a graphene nanosheet (GNS)-filled polymer. The addition of GNSs inside the polymer matrix led to an improvement in the elastic properties, transverse coefficient of thermal expansion, and piezoelectric coefficients *e*_31_ and *e*_15_. The mechanical and piezoelectric characteristics of piezoelectric fiber–CNT-reinforced nanocomposites were investigated by Hasanzadeh et al. [18]. CNTs were randomly oriented into a polymer matrix. The mechanical properties and piezoelectric coefficient $e_{31}$ of the piezoelectric nanocomposite containing CNTs were improved over those of the piezoelectric composite without CNTs [18]. Godara and Mahato [19] studied the elastic and piezoelectric coefficients of a nanocomposite in which piezoelectric fibers were embedded into a CNT-reinforced polymer [19]. The use of CNTs in piezoelectric-fiber-reinforced composites can enhance the structural/functional properties [19].

Generally, evaluating the engineering constants of piezoelectric fibrous nanocomposites containing silica nanoparticles is crucial in designing structures constructed with these materials. Many microstructural factors such as the amount, size, dispersion quality, variation in properties, and nanoparticle–matrix interfacial interaction affect the overall properties of silica-nanoparticle-containing composites [20,21,22]. Therefore, conducting studies in this area is of significance [9,18,23,24,25,26].

To the best of the authors’ knowledge, the piezoelectro-elastic properties of piezoelectric fiber–nanoparticle–polymer nanocomposites with regard to the agglomeration and size of the silica nanoparticles and the silica–polymer interphase have not yet been sufficiently investigated. The novelty of this work comes from developing a hierarchical micromechanical method to comprehensively investigate the elastic and piezoelectric constants of PZT-7A–silica–polyimide nanocomposites with variables of important microstructures. So, the current research aims to evaluate the properties of a piezoelectric nanocomposite made of unidirectional piezoelectric fibers embedded in a polyimide matrix with silica nanoparticles. To achieve this, we developed a model using Ji’s approach and the Mori–Tanaka method. After confirming the accuracy of the model, we studied how the volume, size, and clumping of nanoparticles, as well as the thickness and stiffness of the silica–polymer interface, affect the composite’s elastic and shear moduli and piezoelectric coefficients (*e*_31_ and *e*_33_). One potential use for these nanocomposites is in energy-harvesting devices.

## 2. Micromechanical Analysis of Piezoelectric Fibrous Nanocomposites

**Figure 1:**
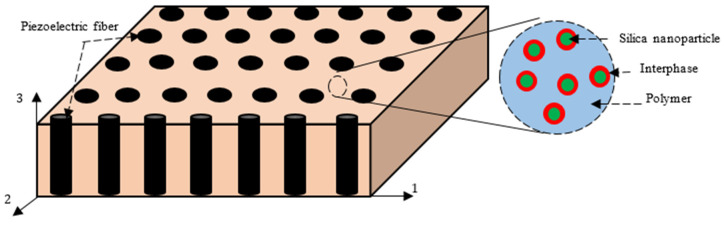
Demonstration of the piezoelectric fibrous nanocomposite with a silica-nanoparticle-filled polymer matrix.

The nanocomposite consists of unidirectional piezoelectric microfibers, silica nanoparticles, and a polymer matrix. Figure 1 shows a representation of a lamina made of this piezoelectric fibrous nanocomposite. The configuration of such a nanocomposite is such that piezoelectric fibers are embedded inside a nanoparticle-filled polymer. Note that the piezoelectric fibers are aligned along the 3-direction. The dispersion of the silica nanoparticles into the polymer matrix can be uniform or non-uniform. The interphase region shown in this figure is considered due to the interaction between the nanoparticles and the polymer matrix. In general, the representative volume element (RVE) of the nanocomposite system may be treated as consisting of two phases, in which the reinforcement is the piezoelectric fiber and the matrix is the nanoparticle-filled polymer. The micromechanical modeling to predict the effective properties of piezoelectric fibrous nanocomposites is carried out here in a two-step procedure. First, the elastic properties of the silica-nanoparticle-filled polymer are calculated using the Ji and Mori–Tanaka models. Next, the Mori–Tanaka model is employed to predict the overall piezoelectric coefficients and elastic moduli of the piezoelectric fibrous nanocomposite.

### 2.1. Silica-Nanoparticle-Filled Polymer

In this sub-section, a micromechanics approach is presented to estimate the effective properties of the polymer matrix containing silica nanoparticles. Micromechanical models predict the composite properties in terms of the volume fraction, geometry of fillers, and material properties of constituents [27,28,29,30]. Concerning the nanoparticle-filled polymer systems, it is required to incorporate the interphase between the nanoparticle and the polymer matrix in the simulation [20,29,31,32,33]. The interfacial region possesses material properties in between those of the polymer matrix and those of the nanoparticle [20,22,31,32,34]. In the micromechanical simulation, the interphase is considered as the third phase, which surrounds the silica nanoparticles, as shown in Figure 1. The Young’s modulus of the silica-filled polymer materials with the interphase can be estimated by the Ji model [35,36] as follows:

(1) $$E_{NC}=E_{m}{\left[\left(1-\lambda \right)+\frac{\lambda -\beta }{\left(1-\lambda \right)+\frac{\lambda (k-1)}{\ln k}}+\frac{\beta }{\left(1-\lambda \right)+\frac{\left(\lambda -\beta )(k+1\right)}{2}+\beta \frac{E_{NP}}{E_{m}}}\right]}^{-1}$$

in which $E_{m}$ and $E_{NP}$ are the Young’s moduli of the matrix and nanoparticle, respectively. The values of λ, β, and k are given by

(2) $$\lambda =\sqrt{{\left(\frac{{2t}_{i}}{d_{NP}}+1\right)}^{3}\varphi_{NP}},\;\beta =\sqrt{\varphi_{NP}},\;k=\frac{E_{i}}{E_{m}}$$

where $\varphi_{NP}$ and $d_{NP}$ are the volume fraction and diameter of the nanoparticle, and $E_{i}$ and $t_{i}$ are the Young’s modulus and thickness of the interphase, respectively. The Young’s modulus of the silica-filled polymer materials in the absence of the interphase, i.e., a two-phase composite, is [36]

(3) $$E_{NC}=E_{m}{\left[\left(1-\beta \right)+\frac{\beta }{\left(1-\beta \right)+\beta \frac{E_{NP}}{E_{m}}}\right]}^{-1}$$

Poisson’s ratio of a nanoparticle-filled polymer can be estimated by the rule of the mixture as $v_{NC}=v_{NP}\varphi_{NP}+v_{m}(1-\varphi_{NP})$, in which $v_{NP}$ and $v_{m}$ are Poisson’s ratio of the nanoparticle and matrix, respectively.

Silica nanoparticles tend to form agglomerates, which leads to poor distribution into the polymer matrix during the fabrication process [20,37,38]. The non-uniform distribution and formation of nanoparticle agglomeration may be mostly attributed to their great specific surface area and high surface energy [37,38]. A two-parameter micromechanical technique is employed to investigate the effect of nanoparticle agglomeration on the effective properties of the nanoparticle-filled polymer [39,40,41]. A number of nanoparticles are supposed to uniformly disperse into the polymer, and other nanoparticles appear in the agglomeration state. The whole volume of the nanoparticles in the RVE of the filled polymer specified by $V_{NP}$ is divided into the following two parts:

(4) $$V_{NP}=V_{NP}^{agg}+V_{NP}^{em}$$

where $V_{NP}^{agg}$ is the volume of nanoparticles inside the agglomerated phase and $V_{NP}^{em}$ denotes the volume of nanoparticles in the polymer matrix and outside the agglomerates. The definition of these two parameters for the agglomeration is as follows:

(5) $$\xi =\frac{V_{agg}}{V},\;\zeta =\frac{V_{NP}^{agg}}{V_{NP}}$$

where V is the volume of the filled polymer RVE and $V_{agg}$ denotes the volume of the agglomerate phase within the RVE [39,40,41]. Thus, the volume fraction of nanoparticles in the agglomeration phase $\varphi_{NP}^{agg}$ and in the polymer matrix and outside the agglomerates $\varphi_{NP}^{em}$ is determined as

(6) $$\varphi_{NP}^{agg}=\frac{V_{NP}^{agg}}{V_{agg}}=\frac{\zeta }{\xi }\varphi_{NP},\;\varphi_{NP}^{em}=\frac{V_{NP}-V_{NP}^{agg}}{{V-V}_{agg}}=\frac{1-\zeta }{1-\xi }\varphi_{NP}$$

We analyze the nanoparticle-filled polymer as a system consisting of agglomerates of spherical shape embedded in a new matrix. We initially predict Young’s modulus and Poisson’s ratio of the agglomerate and the new matrix phases by the Ji model and the rule of mixture. The Young’s modulus and Poisson’s ratio are then used to calculate the bulk modulus and shear modulus. Using the bulk moduli and shear moduli of the agglomerate ($K_{agg}$, $G_{agg}$) and the new matrix ($K_{em}$, $G_{em}$), the equivalent bulk modulus (${\bar{K}}_{NC}$) and shear modulus (${\bar{G}}_{NC}$) of the nanoparticle-filled polymer system with the nanoparticle agglomeration are predicted by the Mori–Tanaka model [39,40,41], respectively,

(7) $${\bar{K}}_{NC}=K_{em}+\frac{\xi \left(\frac{K_{agg}}{K_{em}}-1\right)}{1+\frac{1-\xi }{1+\frac{4G_{em}}{3K_{em}}}\left(\frac{K_{agg}}{K_{em}}-1\right)}K_{em},$$

(8) $${\bar{G}}_{NC}=G_{em}+\frac{\xi \left(\frac{G_{agg}}{G_{em}}-1\right)}{1+\frac{\left(6+12\frac{G_{em}}{K_{em}}\right)}{\left(15+20\frac{G_{em}}{K_{em}}\right)}\left(1-\xi \right)\left(\frac{G_{agg}}{G_{em}}-1\right)}G_{em}.$$

Thus, the elastic properties of a nanoparticle-filled polymer considering the interphase region and agglomeration of nanoparticles can be achieved by the micromechanical technique developed in this section.

### 2.2. Piezoelectric Fiber Composites

Now, a micromechanical model can be adopted to estimate the equivalent properties of piezoelectric fibrous nanocomposites. By the means of the conventional indicial notation, the constitutive equations of piezoelectric materials are as follows [42,43,44]:

(9) $$\sigma_{ij}=C_{ijmn}\varepsilon_{mn}+e_{nij}E_{n}\;D_{i}=e_{imn}\varepsilon_{mn}-k_{in}E_{n}$$

where the repeated sub-scripts are summed over the range of i, j,m,n= 1, 2, 3, and $\sigma_{ij}$ and $\varepsilon_{mn}$ stand for the stress and strain tensors, respectively. $E_{n}$ and $D_{i}$ stand for the electric field and the electric displacement vectors, respectively. $C_{ijmn}$, $e_{nij}$, and $k_{in}$ denote the elastic stiffness and piezoelectric and permittivity tensors, respectively. Divergence equations expressing the mechanical equilibrium and Gauss’ law can be given by Equation (10) [44], respectively:

(10) $$\sigma_{ij,j}=0\;D_{i,i}=0$$

Moreover, the gradient equations defining the strain displacement equations and electric field potential are expressed, respectively:

(11) $$\varepsilon_{ij}=\frac{1}{2}\left(u_{i,j}+u_{j,i}\right)\;E_{i}=-\phi_{,i}$$

in which $u_{i}$ and ϕ stand for the mechanical displacement and electric potential, respectively.

The components of piezoelectric fibrous nanocomposites are the nanoparticle-filled polymer as the matrix phase and the piezoelectric fibers as the reinforcing phase. On the basis of the Mori–Tanaka micromechanical approach and considering $v_{m}$ and $v_{r}$ as the volume fraction of the matrix and fiber, respectively, the electro-elastic constants of piezoelectric fibrous nanocomposites can be estimated as

(12) $${\tilde{C}}^{c}={\tilde{C}}^{m}+v_{r}\left({\tilde{C}}^{r}-{\tilde{C}}^{m}\right)B$$

in which ${\tilde{C}}^{r}$ and ${\tilde{C}}^{m}$ are the electro-elastic modulus matrices for the reinforcement and matrix, respectively, and the piezoelectric concentration tensor is defined as follows:

(13) $$B=A{\left[v_{m}I+v_{r}A\right]}^{-1},A={\left[I+\hat{S}({{\tilde{C}}^{m})}^{-1}\left({\tilde{C}}^{r}-{\tilde{C}}^{m}\right)\right]}^{-1},$$

where $\hat{S}$ is the Eshelby tensor and its components can be found in the literature [18,24,27,44]. It is worth mentioning that all simulation procedures and numerical results obtained in this research have been performed using codes written in MATLAB(R2024b) software. Also, it should be noted that all formulations used for the simulations in MATLAB software are analytical relations, and the finite element method has not been employed.

## 3. Results and Discussion

In this section, we first present the numerical results of the Young’s moduli, shear moduli, and piezoelectric coefficients of PZT-7A fiber-reinforced nanocomposites with the silica-nanoparticle-filled polyimide matrix. Then, some comparisons are made between the present predictions and other results available in the literature [45,46].

### 3.1. Piezoelectro-Elastic Response of Piezoelectric Fibrous Nanocomposites

The micromechanical model consisting of Ji’s model, the rule of mixture, and the Mori–Tanaka model is used to investigate the effective constants of the piezoelectric fibrous nanocomposite. The constituents of the composite are PZT-7A, polyimide, and silica nanoparticles. The Young’s modulus and Poisson’s ratio of the polyimide and silica nanoparticles are 3.78 GPa and 0.4 and 73 GPa and 0.23 [20,22,44], respectively. Table 1 lists the properties of the PZT-7A fiber. The piezoelectric fiber volume fraction is considered to be 60%. The diameter of the silica nanoparticles and their volume fraction into the polyimide matrix are 30 nm and 3%, respectively. Also, the Young’s modulus, Poisson’s ratio, and thickness of the interphase region are taken as $E_{i}=10E_{m}$, 0.4, and $t_{i}=0.5d_{NP}$ [20,31], respectively.

**Figure 2:**
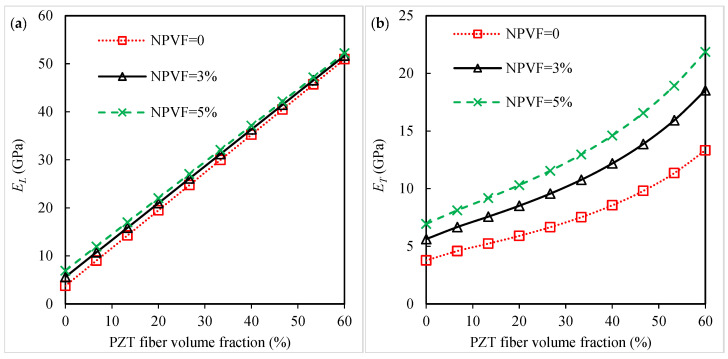

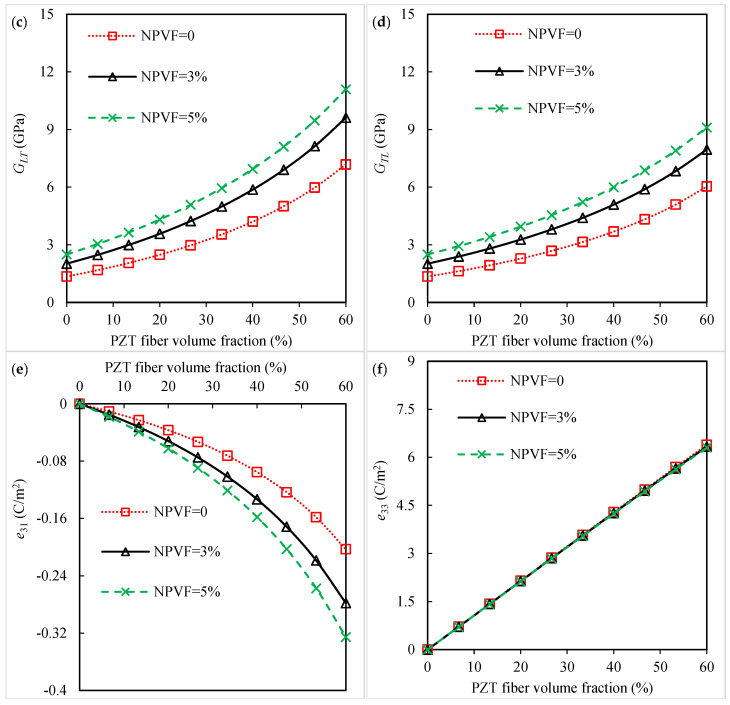
Influence of nanoparticle volume fraction on the (**a**) longitudinal Young’s modulus, (**b**) transverse Young’s modulus, (**c**) longitudinal shear modulus, (**d**) transverse shear modulus, (**e**) piezoelectric coefficient *e*_31_, and (**f**) piezoelectric coefficient *e*_33_ of the piezoelectric fibrous nanocomposite containing silica nanoparticles.

Figure 2 shows the influence of adding silica nanoparticles on the material constants of the piezoelectric fibrous nanocomposites. The numerical results are presented for two values of nanoparticle volume fraction (NPVF), 3% and 5%. The material properties of piezoelectric fibrous composites without nano-inclusions are also illustrated in the figure. Figure 2a indicates the results of the longitudinal Young’s modulus versus piezoelectric fiber volume fraction. Incorporating nano-inclusions into the polymer matrix insignificantly affects the longitudinal Young’s modulus. This is attributed to the fact that the longitudinal properties of long fiber-reinforced composites are mostly dominated by the material properties and content of fibers [9,18,47,48]. It can be seen in Figure 2a that Young’s modulus in the direction parallel to the fiber direction linearly increases as the piezoelectric fiber volume fraction increases. Figure 2b shows the variation in the transverse Young’s modulus of the piezoelectric fibrous nanocomposites with the piezoelectric fiber volume fraction. The transverse Young’s modulus notably depends on the nanoparticles in the polyimide matrix. The value of $E_{T}$ of the silica-nanoparticle-containing nanocomposite is greater than that of the composite without silica nanoparticles. When the piezoelectric fiber volume fraction is 60%, the nanocomposite with a 3% silica-nanoparticle-containing polyimide exhibits a 39% improvement in the transverse Young’s modulus in comparison with the composite without nanoparticles. Adding silica nanoparticles within the polyimide provides a relatively stronger matrix that is potentially beneficial for the transverse Young’s modulus of the piezoelectric fibrous nanocomposites. The increase in the silica nanoparticle amount aids the nanocomposite in obtaining a higher value of the transverse Young’s modulus. A similar trend has been found for other types of nanofillers [9,18,48]. It is shown in Figure 2b that the Young’s modulus in the direction perpendicular to the piezoelectric fiber nonlinearly increases as the fiber volume fraction increases. Based on the results observed in Figure 2c,d, adding silica nanoparticles is generally beneficial to shear moduli in the longitudinal and transverse directions. Both shear moduli are enhanced by increasing the piezoelectric fiber volume fraction. The values of the transverse shear modulus of the 60% piezoelectric-fiber-reinforced composite without nanoparticles and with a 3% nanoparticle content are calculated as 6.04 GPa and 7.96 GPa, respectively. Figure 2e depicts the piezoelectric coefficient $e_{31}$ of the piezoelectric fibrous nanocomposites versus the piezoelectric fiber volume fraction. The value of piezoelectric coefficient $e_{31}$ can be significantly improved as a result of the nanoparticle addition into the polyimide matrix. A major contribution to the piezoelectric coefficient $e_{31}$ is from the polymer matrix properties. Compared to the piezoelectric fibrous composite, the piezoelectric coefficient $e_{31}$ of the piezoelectric fibrous nanocomposite containing a 3% silica-nanoparticle-filled polyimide matrix exhibits an upward trend with an approximate 37% improvement. Therefore, incorporating nanofillers into the polymer can enhance the in-plane actuation property of piezoelectric fibrous nanocomposites over that of a traditional piezoelectric fibrous composite without nanofillers. This trend has been reported for other composite systems containing CNTs [9,18,19]. The piezoelectric coefficient $e_{31}$ of piezoelectric fibrous nanocomposites exhibits an improvement through the increase in the piezoelectric fiber volume fraction. Figure 2f displays the piezoelectric coefficient $e_{33}$ versus the fiber volume fraction. There is no effect of silica nanoparticles on the piezoelectric coefficient $e_{33}$ because its value is mostly dominated by the piezoelectric property of the fiber. A linear increase is obtained for the piezoelectric coefficient $e_{33}$ as the piezoelectric fiber volume fraction increases. Because the piezoelectric constant $e_{31}$ and the elastic properties such as $E_{T}$ and $G_{TL}$ of the piezoelectric fibrous nanocomposite containing silica nanoparticles are improved, this composite has good potential for use as a superior actuator material for intelligent structures with a great in-plane actuation option [9,12].

**Figure 3:**
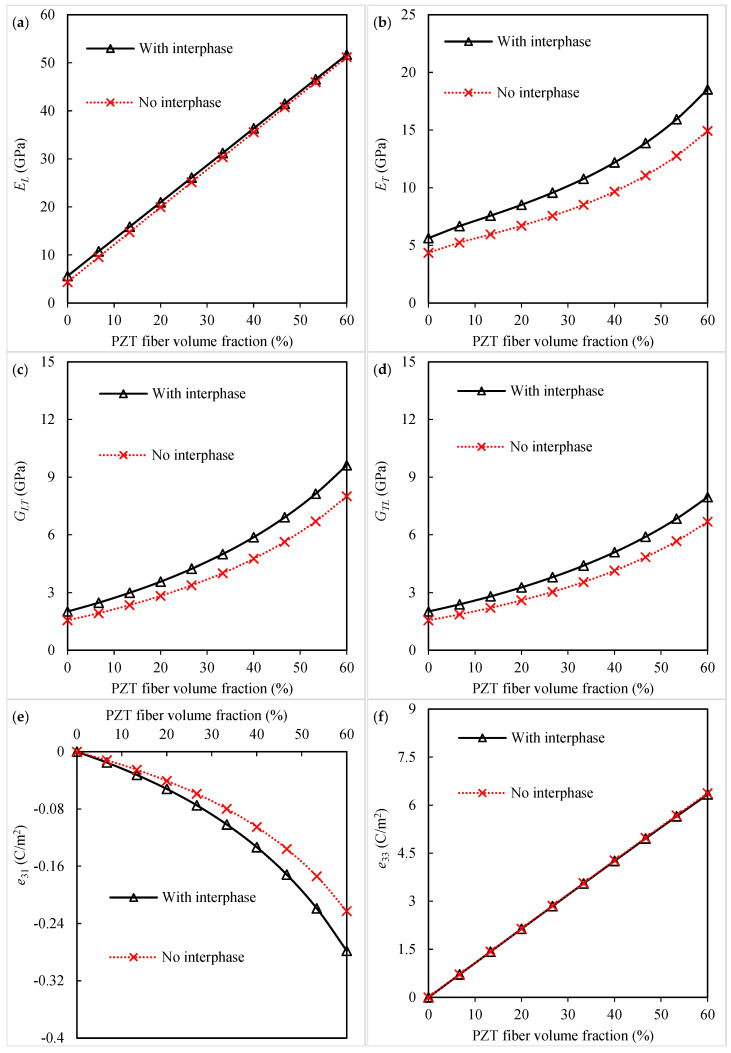
Influence of interphase region on the (**a**) longitudinal Young’s modulus, (**b**) transverse Young’s modulus, (**c**) longitudinal shear modulus, (**d**) transverse shear modulus, (**e**) piezoelectric coefficient *e*_31_, and (**f**) piezoelectric coefficient *e*_33_ of the piezoelectric fibrous nanocomposite containing silica nanoparticles.

The micromechanical results for investigating the role of the interfacial zone between the silica nanoparticles and polyimide matrix in the material constants of the piezoelectric fibrous nanocomposites are presented in Figure 3. It is worth pointing out that the interphase does not have a notable contribution to the Young’s modulus in the longitudinal direction, as seen in Figure 3a. It may be concluded from Figure 3b–d that the formation of the interfacial region is beneficial to the transverse Young’s modulus and both shear moduli. Relative to the composite system without the interphase, an increasing trend is observed for these three elastic moduli of the nanocomposite with the interphase. The results of Figure 3e disclose that the interphase tends to improve the piezoelectric coefficient $e_{31}$. A literature survey shows that the interphase between the polymer matrix and CNTs can contribute to the improvement of the overall properties of piezoelectric–CNT nanocomposites [18]. As shown in Figure 3f, the piezoelectric coefficient $e_{33}$ exhibits no variation in the presence or absence of the interphase.

**Figure 4:**
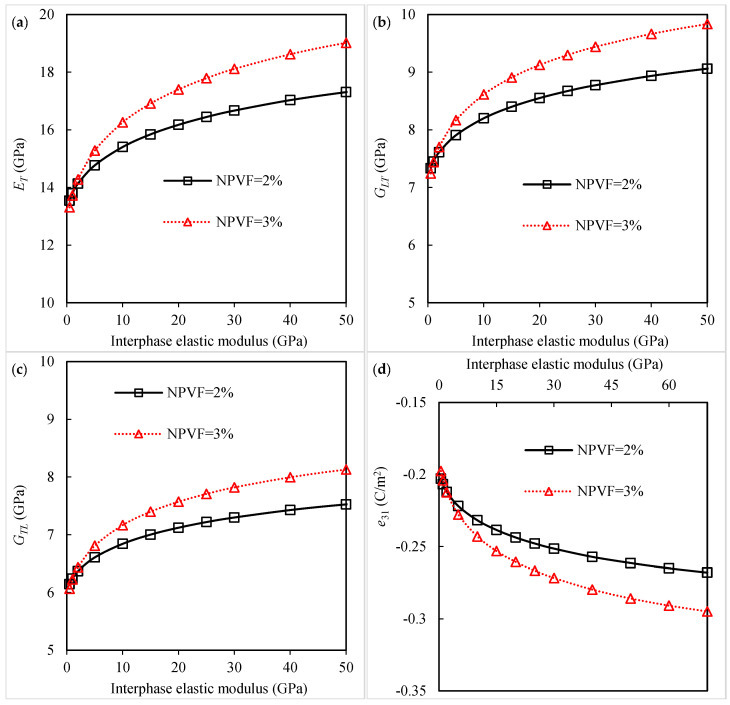
Variation in (**a**) transverse Young’s modulus, (**b**) longitudinal shear modulus, (**c**) transverse shear modulus, and (**d**) piezoelectric coefficient *e*_31_ of piezoelectric fibrous nanocomposite with interphase elastic modulus.

Generally, the interphase has properties in between those of the nanoparticle and those of the polymer matrix [20,22,29,31,32,34]. To better evaluate the effect of interphase characteristics on the material constants of piezoelectric fibrous nanocomposites, a micromechanical analysis is conducted with different values of interphase stiffness and thickness. The results of the transverse Young’s modulus, longitudinal shear modulus, transverse shear modulus, and piezoelectric coefficient $e_{31}$ with changing the interphase elastic modulus are shown in Figure 4a–d, respectively. The effective properties of the piezoelectric fibrous nanocomposite can be enhanced with increasing the interphase elastic modulus. It is noted that a stiffer interphase can increase the mechanical properties of the polymer matrix [18,20,22,29]. One of the ways to enhance the mechanical properties of the interfacial region may be nanoparticle surface treatment.

**Figure 5:**
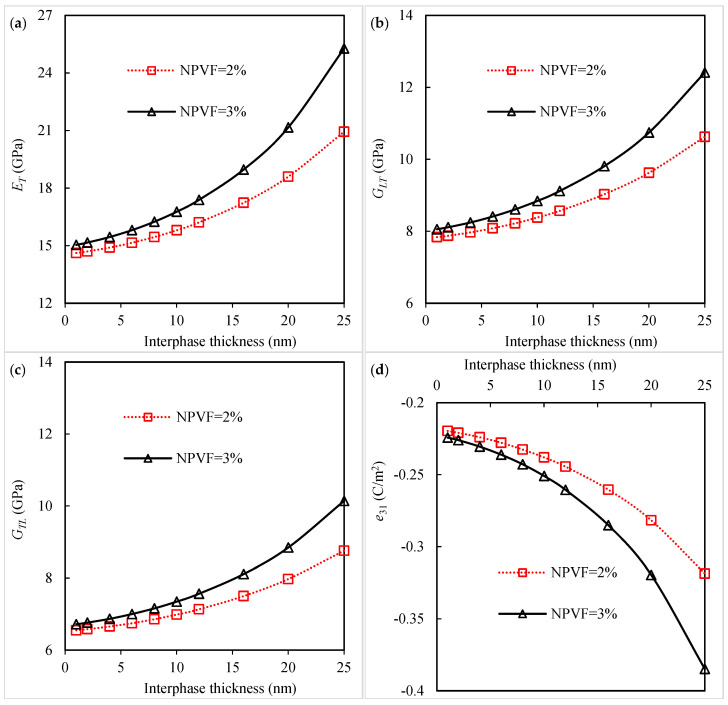
Variation in (**a**) transverse Young’s modulus, (**b**) longitudinal shear modulus, (**c**) transverse shear modulus, and (**d**) piezoelectric coefficient *e*_31_ of piezoelectric fibrous nanocomposite with interphase thickness.

The influence of changing the interphase thickness on the properties of nanocomposites, including $E_{T}$, $G_{LT}$, $G_{TL}$, and $e_{31}$, is depicted in Figure 5a–d, respectively. The increase in interphase thickness significantly improves the elastic and piezoelectric constants. Different methods for nanoparticle functionalization may produce interphases with variable thicknesses. By increasing the interphase thickness from 1 nm to 15 nm, the improvement in the transverse elastic modulus with a 3% silica-nanoparticle-containing polyimide matrix is calculated to be about 20.5%. In turn, for the piezoelectric coefficient $e_{31}$, the improvement is about 26%. It is worth mentioning that the in-plane actuation caused by the piezoelectric composite is increased by tailoring the piezoelectric constant $e_{31}$ [9,47]. An important conclusion from the above micromechanical studies is the production of a stiff and thick interphase in the nanocomposite fabrication. This is due to the increased stiffness of the polymer matrix by adding nanoparticles, as reported in previous studies [18,20,22,29].

**Figure 6:**
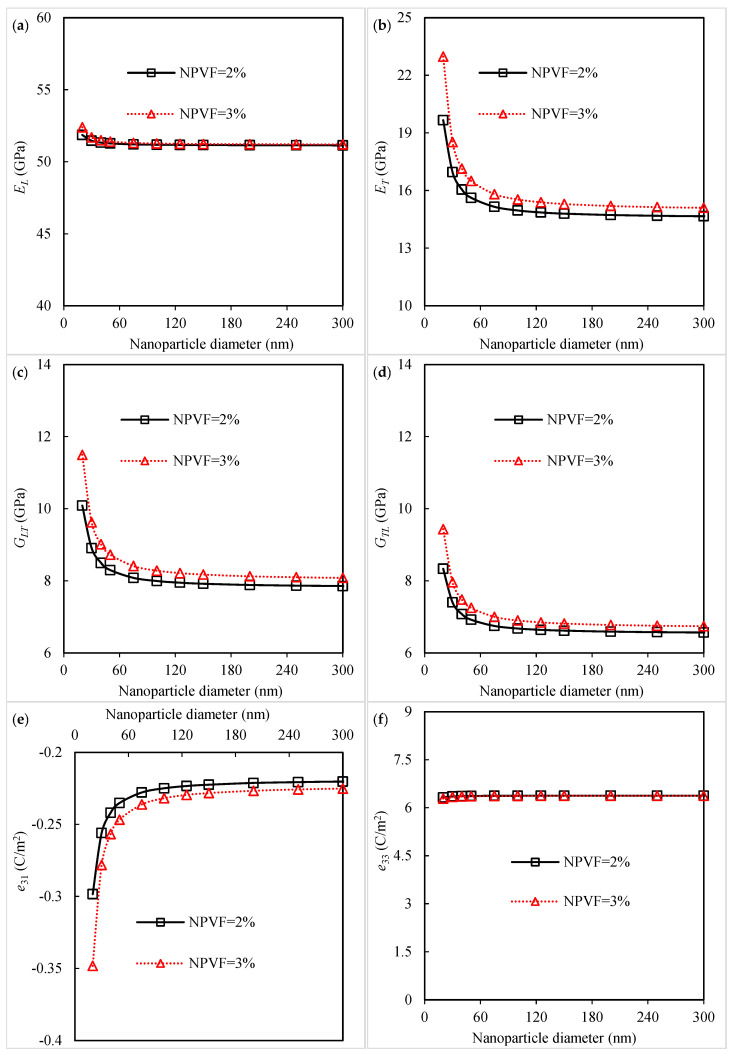
Influence of silica nanoparticle diameter on the (**a**) longitudinal Young’s modulus, (**b**) transverse Young’s modulus, (**c**) longitudinal shear modulus, (**d**) transverse shear modulus, (**e**) piezoelectric coefficient *e*_31_, and (**f**) piezoelectric coefficient *e*_33_ of the piezoelectric fibrous nanocomposite containing silica nanoparticles.

The influence of the silica nanoparticle diameter on the mechanical properties and piezoelectric coefficients of piezoelectric fibrous nanocomposites is studied, and the results are displayed in Figure 6. As seen in Figure 6a, the values for the longitudinal Young’s modulus with different nanoparticle diameters are close to each other, indicating the insignificant contribution of nanoparticle size to this elastic property. It is seen from Figure 6b that the addition of uniformly dispersed silica nanoparticles with smaller sizes results in an increase in the Young’s modulus in the transverse direction. This may be explained by the interphase contribution to the final properties of nanocomposites becoming more prominent as the nanoparticle size decreases [20,48]. A notable enhancement in the shear moduli along both the longitudinal and transverse directions can be observed by the decrease in nanoparticle size, as shown in Figure 6c,d. The results of Figure 6e disclose that a smaller size of silica nanoparticles is required so as to further improve the piezoelectric coefficient $e_{31}$ of the nanocomposites. In the case of the nanocomposite with a 3% silica-nanoparticle-containing polyimide matrix, the improvement is about 50% by decreasing the nanoparticle diameter from 100 nm to 20 nm. As $d_{NP}>$ 100 nm, the change in nanoparticle diameter does not affect the elastic moduli or the piezoelectric coefficient $e_{31}$. The main reason for this behavior may be the reduced effect of the interphase. As the size of the nanoscale particles increases and goes to the microscale, the role of the interphase in the effective properties of the nanocomposites decreases. According to the outcomes of Figure 6f, the piezoelectric coefficient $e_{33}$ of the nanocomposites does not depend on the nano-inclusion size since the piezoelectric fibers have the main role in this property. Thus, the hybridization of the piezoelectric fibers with smaller nanoparticles induces better elastic moduli $E_{T}$, $G_{LT}$, and $G_{TL}$ and a better piezoelectric coefficient $e_{31}$.

**Figure 7:**
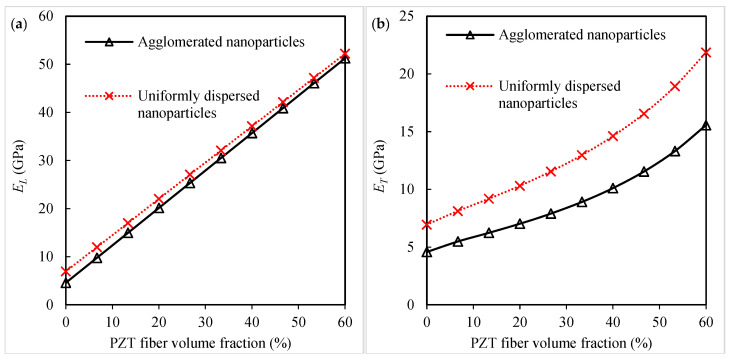

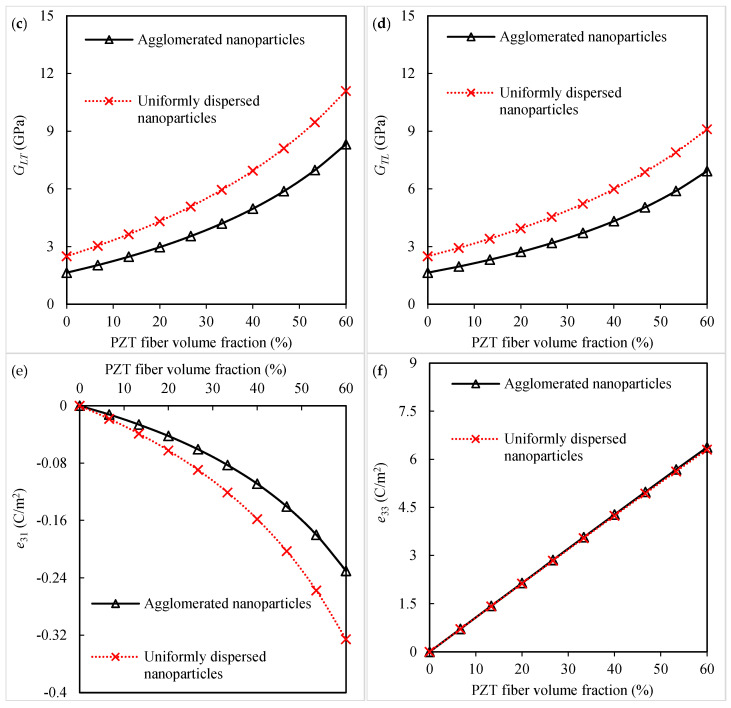
Influence of silica nanoparticle dispersion quality on the (**a**) longitudinal Young’s modulus, (**b**) transverse Young’s modulus, (**c**) longitudinal shear modulus, (**d**) transverse shear modulus, (**e**) piezoelectric coefficient *e*_31_, and (**f**) piezoelectric coefficient *e*_33_ of the piezoelectric fibrous nanocomposite containing silica nanoparticles.

To study the effect of dispersion quality of silica nanoparticles, the elastic moduli and piezoelectric coefficients of the piezoelectric fibrous nanocomposites are calculated for two conditions, including a uniform dispersion and an agglomerated state (ζ=0.9, ξ=0.1). The numerical results of the micromechanical analysis are presented in Figure 7a–f. In this sensitivity study, the nanoparticle volume fraction is 5%. It is observed from Figure 7a that the longitudinal Young’s modulus is minimally affected by the non-uniform dispersion of the nanoparticles. The other three elastic constants, the transverse Young’s modulus, longitudinal shear modulus, and transverse shear modulus, appear to significantly decrease due to the formation of silica nanoparticle agglomeration (Figure 7b–d). The agglomeration of the silica nanoparticles produces a negative effect on the piezoelectric coefficient $e_{31}$. As compared to the uniformly dispersed case, a decrease of about 29.2% in the piezoelectric coefficient $e_{31}$ is observed by forming the agglomeration. As mentioned in previous studies [20,37,38,48], nanoparticle agglomeration leads to a reduction in the mechanical properties of polymer matrix nanocomposites. The nanocomposite containing uniformly dispersed silica nanoparticles exhibits a higher piezoelectric coefficient $e_{31}$ than that containing agglomerated nanoparticles. It is shown in Figure 7f that the dispersion quality does not affect the estimated piezoelectric coefficient $e_{33}$. Uniformly dispersing and avoiding the agglomeration of nanoparticles into the polymer matrix are critical for advanced composite materials to take the maximum material constants, i.e., $E_{T}$, $G_{LT}$, $G_{TL}$, and $e_{31}$.

**Figure 8:**
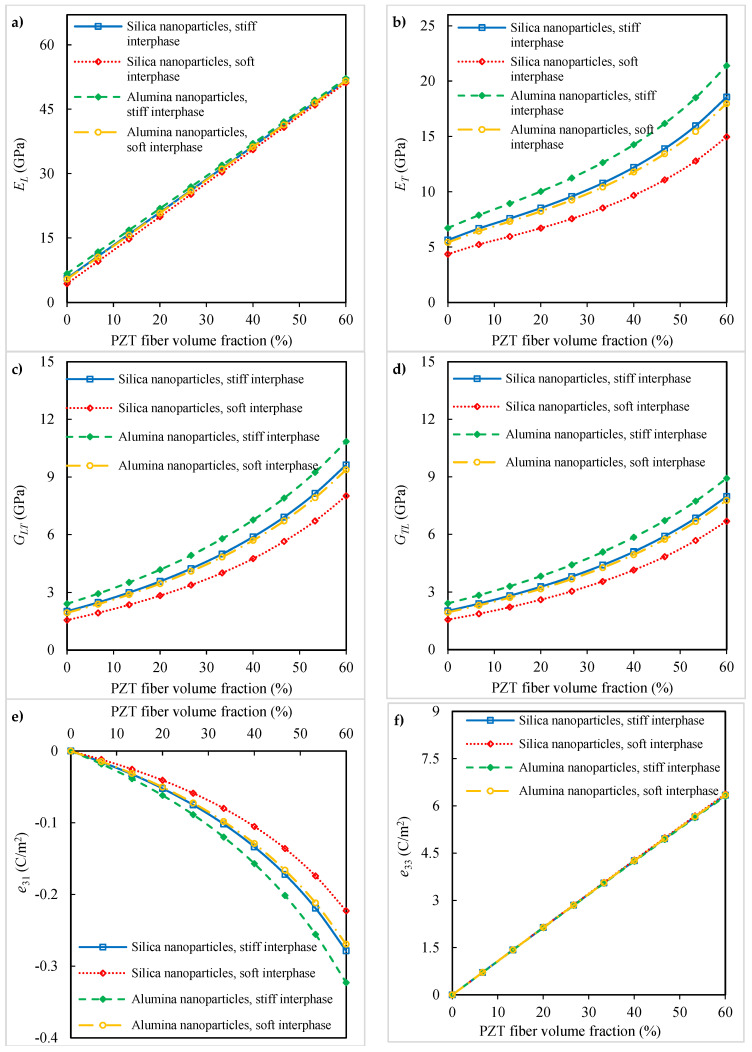
Influence of nanoparticle types on the (**a**) longitudinal Young’s modulus, (**b**) transverse Young’s modulus, (**c**) longitudinal shear modulus, (**d**) transverse shear modulus, (**e**) piezoelectric coefficient *e*_31_, and (**f**) piezoelectric coefficient *e*_33_ of the piezoelectric fibrous nanocomposite containing nanoparticles.

The results of the micromechanical analysis with silica and alumina nanoparticles individually incorporated into the polyimide matrix are presented in Figure 8. The volume fraction and diameter for both nanoparticles are identical. The influence of the interphase stiffness on the effective properties of the piezoelectric fibrous nanocomposites is also examined. The change in the interphase stiffness is taken in a range from the soft material to the stiff material. The soft interphase ($E_{i,soft}$) can be categorized as the material having very low stiffness in comparison with the reinforcement stiffness [49] as

(14) $$E_{i,soft}=\frac{E_{NP}+E_{m}}{20}$$

The stiff interphase ($E_{i,stiff}$) can be categorized as the material having an average value of reinforcement and matrix stiffness and is very high in comparison with the matrix stiffness [49] as

(15) $$E_{i,stiff}=\frac{E_{NP}+E_{m}}{2}$$

On the basis of the outcomes shown in Figure 8a,f, the incorporation of different types of nano-inclusions has a negligible effect on the longitudinal Young’s modulus and piezoelectric coefficient $e_{33}$. In contrast, changes in the nano-inclusion type embedded within the matrix affect the elastic moduli $E_{T}$, $G_{LT}$, and $G_{TL}$ and the piezoelectric coefficient $e_{31}$ of the nanocomposites, as shown in Figure 8b–e. Compared to the silica nanoparticles, the use of alumina nanoparticles in the polyimide matrix can further improve these material properties. Again, nanocomposites with a stiff interphase show higher elastic moduli $E_{T}$, $G_{LT}$, and $G_{TL}$ and a higher piezoelectric coefficient $e_{31}$ than those with a soft interphase. Due to the good mechanical and piezoelectric properties, the piezoelectric fiber–nanoparticle–polymer nanocomposites can find various industrial applications, such as in actuators, sensors, and energy-harvesting devices [9,12,26].

### 3.2. Comparisons with Experimental and Numerical Results

**Figure 9:**
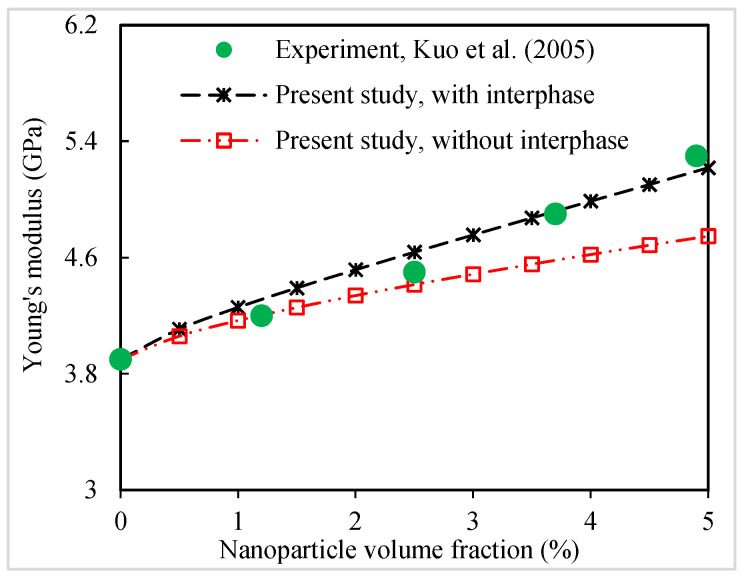
Present predictions for Young’s modulus of silica-nanoparticle-filled PEEK nanocomposites compared to experimental data [45].

Now, the predictions are compared with the available experimental data of some silica-filled polymer composites for validating the micromechanical model. Figure 9 presents a comparison between the present predictions and experimental data [45] of the Young’s modulus of silica-nanoparticle-filled poly(ether-ether-ketone) (PEEK) nanocomposites. Micromechanical tests are carried out in two different states: (1) in the presence of an interphase with k=5, $t_{i}=0.25\times d_{NP}$ and (2) in the absence of an interphase. The silica nanoparticles with a mean diameter ~30 nm are uniformly dispersed in the polymer matrix. The Young’s moduli of the silica nanoparticle and PEEK matrix are 73 GPa and 3.9 GPa, respectively. Figure 9 shows that the model without the interphase agrees better with the experiments for lower values of nanoparticle volume fraction. However, at higher nanoparticle contents, the model predictions with the interfacial region between the nanoparticles and polymer matrix give a more reasonable agreement as compared to the experiments [45]. The interphase region with a higher Young’s modulus than that of the matrix material increases the Young’s modulus of the nanocomposites significantly.

**Figure 10:**
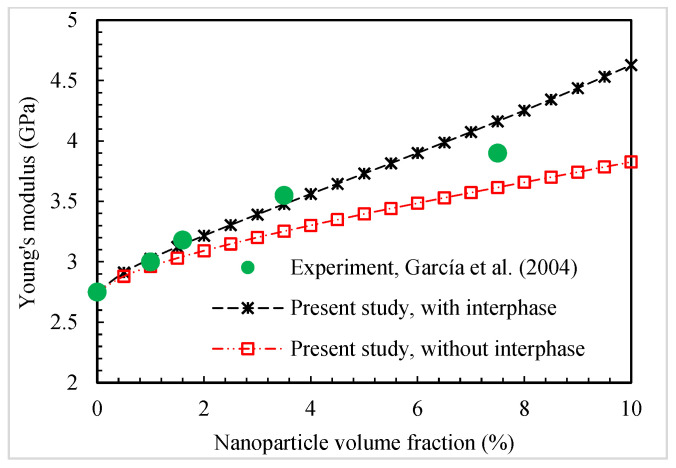
Present predictions for Young’s modulus of silica-nanoparticle-filled nylon-6 nanocomposites compared to experimental data [46].

The Young’s modulus of the silica-nanoparticle-filled nylon-6 nanocomposites determined by the present micromechanics method are compared with the experimental data [46]. Figure 10 shows the outcome of this comparison. The effect of considering the interphase in the micromechanical modeling on the final elastic modulus is also examined. It is observed that the two sets of results evaluated by the micromechanical model by taking the interphase region and the experimental route are in a good agreement.

**Figure 11:**
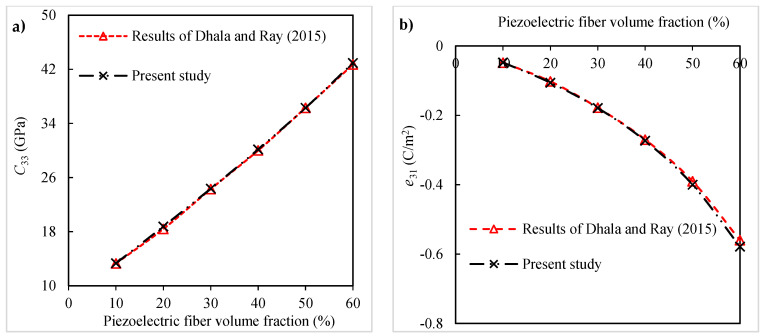
Comparison between the results of the present model and results of [9] for (**a**) elastic constant $C_{33}$ and (**b**) piezoelectric coefficient *e*_31_ of PZT5-fiber-reinforced epoxy composites.

In another comparison, two effective properties of the PZT5-fiber-reinforced epoxy composite including the elastic constant $C_{33}$ and piezoelectric coefficient *e*_31_ predicted by the present micromechanical model are compared with the Mori–Tanaka predictions carried out in [9]. The material constants of the PZT5 fiber as well as the epoxy are given in Table 1 [9,44]. It is shown in Figure 11a,b that the two sets of results are in a very good agreement for both effective constants.

**Figure 12:**
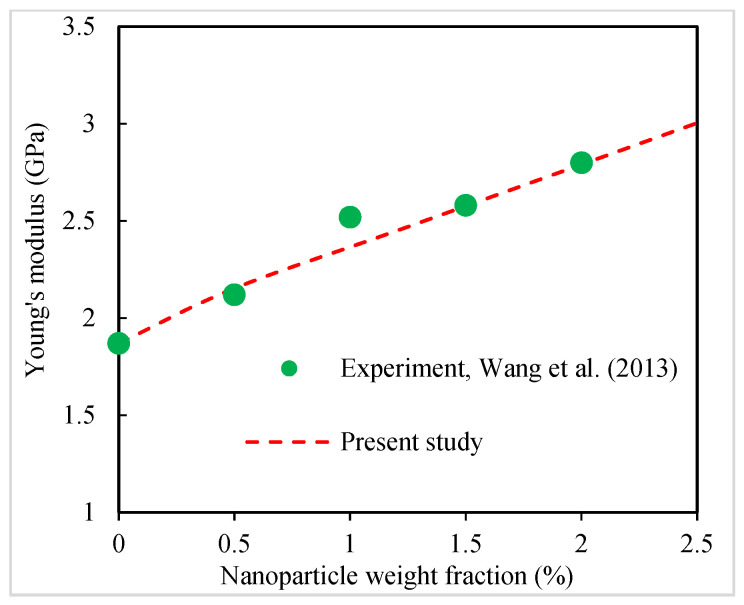
Present predictions for Young’s modulus of silica-nanoparticle-filled P(MMA-co-MTC) nanocomposites compared to experimental data [50].

Wang et al. [50] used silica nanoparticles to produce a polymer nanocomposite: methyl methacrylate (MMA) was chosen as the matrix and copolymerized with a low amount of cationic functional comonomer 2-(methacryloyloxy)ethyltrimethylammonium chloride (MTC). Figure 12 shows another comparison between the present predictions and experimental measurements [50] for the Young’s modulus of silica-nanoparticle-filled P(MMA-co-MTC) nanocomposites. Silica nanoparticles have an average diameter of around 20 nm [50]. A good agreement is observed between the model predictions and the experimental measurements at all nanoparticle contents.

## 4. Conclusions

In this paper, the piezoelectro-elastic coefficients of PZT-7A-fiber-reinforced nanocomposites with a silica-nanoparticle-filled polyimide matrix were evaluated. First, the Ji and Mori–Tanaka models were hierarchically employed to predict the elastic properties of the silica-nanoparticle-filled polymer. The nanoparticle–polymer interphase and the nanoparticle agglomeration were included in the analysis. Then, considering the nanoparticle-filled polymer as the matrix and the piezoelectric fiber as the reinforcement, the Mori–Tanaka model was employed to predict the elastic and piezoelectric constants of the piezoelectric fibrous nanocomposites. A good agreement was observed between the present predictions and other results available in the literature. The results showed that adding silica nanoparticles into the polyimide matrix improves the elastic and piezoelectric properties ($E_{T}$, $G_{LT}$, $G_{TL}$, and $e_{31}$) of the piezoelectric fibrous nanocomposites. As compared to the composite without nanoparticles, 39%, 31.8%, and 37% improvements in the values of $E_{T}$, $G_{TL}$, and the piezoelectric coefficient $e_{31}$ were observed once the volume fractions of the fiber and nanoparticle were 60% and 3%, respectively. More improvement in the elastic moduli $E_{T}$, $G_{LT}$, and $G_{TL}$ and the piezoelectric coefficient $e_{31}$ was found by decreasing the nanoparticle diameter. A thicker and stiffer interphase led to an increase in the elastic moduli $E_{T}$, $G_{LT}$, and $G_{TL}$ and the piezoelectric coefficient $e_{31}$ of the piezoelectric fibrous nanocomposites. However, the nanoparticle agglomeration that formed in the polymer matrix decreased the elastic moduli $E_{T}$, $G_{LT}$, and $G_{TL}$ and the piezoelectric coefficient $e_{31}$. It was observed that increasing the piezoelectric fiber volume fraction increased the piezoelectro-elastic constants of the piezoelectric fibrous nanocomposites.

## Figures and Tables

**Table 1:** Material constants of PZT5 fiber, epoxy, and PZT-7A fiber [9,44].

Material	PZT5	Epoxy	PZT-7A
*C*_11_ (GPa)	121	8	148
*C*_12_ (GPa)	75.4	4.4	76.2
*C*_13_ (GPa)	75.2	4.4	74.2
*C*_22_ (GPa)	121	8	148
*C*_23_ (GPa)	75.2	4.4	74.2
*C*_33_ (GPa)	111	8	131
*C*_44_ (GPa)	21.1	1.8	25.4
*C*_66_ (GPa)	22.8	1.8	35.9
*e*_31_ (C/m^2^)	−5.4	0	−2.1
*e*_33_ (C/m^2^)	9.5	0	9.5

## Data Availability

The original contributions presented in the study are included in the article, further inquiries can be directed to the corresponding author.
